# Radiological and Molecular Analysis of Radioiodinated Anastrozole and Epirubicin as Innovative Radiopharmaceuticals Targeting Methylenetetrahydrofolate Dehydrogenase 2 in Solid Tumors

**DOI:** 10.3390/pharmaceutics16050616

**Published:** 2024-05-03

**Authors:** Mazen Abdulrahman Binmujlli

**Affiliations:** Department of Internal Medicine, College of Medicine, Imam Mohammad Ibn Saud Islamic University (IMSIU), P.O. Box 90950, Riyadh 11623, Saudi Arabia; dr1mazen@gmail.com

**Keywords:** radiopharmaceuticals, MTHFD2, molecular docking, molecular dynamic, MM-PBSA

## Abstract

In the dynamic field of radiopharmaceuticals, innovating targeted agents for cancer diagnosis and therapy is crucial. Our study enriches this evolving landscape by evaluating the potential of radioiodinated anastrozole ([^125^I]anastrozole) and radioiodinated epirubicin ([^125^I]epirubicin) as targeting agents against MTHFD2-driven tumors. MTHFD2, which is pivotal in one-carbon metabolism, is notably upregulated in various cancers, presenting a novel target for radiopharmaceutical application. Through molecular docking and 200 ns molecular dynamics (MD) simulations, we assess the binding efficiency and stability of [^125^I]anastrozole and [^125^I]epirubicin with MTHFD2. Molecular docking illustrates that [^125^I]epirubicin has a superior binding free energy (∆*G*_bind_) of −41.25 kJ/mol compared to −39.07 kJ/mol for [^125^I]anastrozole and −38.53 kJ/mol for the control ligand, suggesting that it has a higher affinity for MTHFD2. MD simulations reinforce this, showing stable binding, as evidenced by root mean square deviation (RMSD) values within a narrow range, underscoring the structural integrity of the enzyme–ligand complexes. The root mean square fluctuation (RMSF) analysis indicates consistent dynamic behavior of the MTHFD2 complex upon binding with [^125^I]anastrozole and [^125^I]epirubicin akin to the control. The radius of gyration (RG) measurements of 16.90 Å for MTHFD2-[^125^I]anastrozole and 16.84 Å for MTHFD2-[^125^I]epirubicin confirm minimal structural disruption upon binding. The hydrogen bond analysis reveals averages of two and three stable hydrogen bonds for [^125^I]anastrozole and [^125^I]epirubicin complexes, respectively, highlighting crucial stabilizing interactions. The MM-PBSA calculations further endorse the thermodynamic favorability of these interactions, with binding free energies of −48.49 ± 0.11 kJ/mol for [^125^I]anastrozole and −43.8 kJ/mol for MTHFD2-. The significant contribution of Van der Waals and electrostatic interactions to the binding affinities of [^125^I]anastrozole and [^125^I]epirubicin, respectively, underscores their potential efficacy for targeted tumor imaging and therapy. These computational findings lay the groundwork for the future experimental validation of [^125^I]anastrozole and [^125^I]epirubicin as MTHFD2 inhibitors, heralding a notable advancement in precision oncology tools. The data necessitate subsequent in vitro and in vivo assays to corroborate these results.

## 1. Introduction

In recent years, there has been substantial progress in the utilization of targeted radiopharmaceuticals for cancer diagnosis and therapy [1,2,3,4,5]. These drugs contain radioactive materials and are designed to selectively deliver radiation to cancer cells, thereby minimizing harm to healthy tissues [1,2,3,4,5]. This targeted approach has demonstrated effectiveness with minimal toxicity when compared to other systemic cancer treatment methods [1,2,3,4,5]. Consequently, there has been a surge in clinical trials and commercial development, with several radiopharmaceutical drugs being approved for clinical use in recent years [6,7]. This emerging treatment approach differs from traditional external radiation therapy and has the potential to revolutionize radiation oncology [8]. The significance of targeted radiopharmaceuticals lies in their capacity to detect and eradicate cancer cells, including small metastatic deposits, offering a promising avenue for cancer diagnosis and treatment [9,10,11].

### 1.1. Tumor Imaging with Radiotracers

Tumor imaging agents, which utilize radiotracers designed to bind to specific cellular receptors, have demonstrated efficacy, thereby affirming the value of receptor-specific targeting in the development of novel radiopharmaceuticals [12,13]. This approach allows for the selective visualization of solid tumors through diagnostic radiopharmaceuticals. A key parameter for these radiopharmaceuticals to function as effective tumor imaging agents is their high target-to-non-target (T/NT) ratio [14]. This ratio quantifies radiopharmaceuticals’ proficiency in directing radiotracers specifically to the receptors present in tumor cells [15]. The existing literature posits that radiopharmaceuticals exhibiting a T/NT ratio exceeding 1.5—indicating a 50% higher accumulation in targeted tissues—hold promise as potential diagnostic tools [16,17]. It is crucial to distinguish, however, that while such a T/NT ratio is suitable for diagnostic purposes, therapeutic applications require a significantly higher T/NT ratio to ensure the therapy’s effectiveness by maximizing the radiation dose to the tumor and minimizing exposure to healthy tissues, thus avoiding undue radiation effects [18,19]. Additionally, an elevated target-to-blood (T/B) ratio is crucial for enhancing the diagnostic efficacy of radiopharmaceuticals in solid tumor imaging [12,17].

Positron emission tomography (PET) plays a pivotal role in the imaging of tumors, offering critical insights into cellular processes and tumor physiology [20,21,22,23]. ^18^F-fluorodeoxyglucose ([^18^F]FDG), the most widely used PET tracer, is fundamental in assessing glucose metabolism in tumors, reflecting its unparalleled importance in oncology [24]. Alongside [^18^F]FDG, radiotracers such as ^18^F-fluoromisonidazole (FMISO), ^18^F-fluoroazomycin arabinoside ([^18^F]FAZA), and ^18^F-flortanidazole ([^18^F]HX4) serve as notable examples of agents targeting hypoxic tumor environments, further expanding the capabilities of PET imaging in cancer diagnosis and management [20,21,22,23]. Despite their utility, the use of ^18^F-labeled compounds is often constrained by significant production costs and a short half-life [25]. In contrast, single-photon emission computed tomography (SPECT), which is widely adopted across many nuclear medicine facilities, has seen significant advancements with the development of radioiodinated and [^99m^Tc]-labeled radiopharmaceuticals targeting solid tumors [26,27,28,29]. These novel agents aim to optimize tumor imaging by enhancing tumor uptake, target-to-non-target (T/NT) ratios, and target-to-blood (T/B) ratios [30]. However, it is important to recognize that the efficacy of these enhancements is marker- and tumor-specific [25].

**Figure 1:**
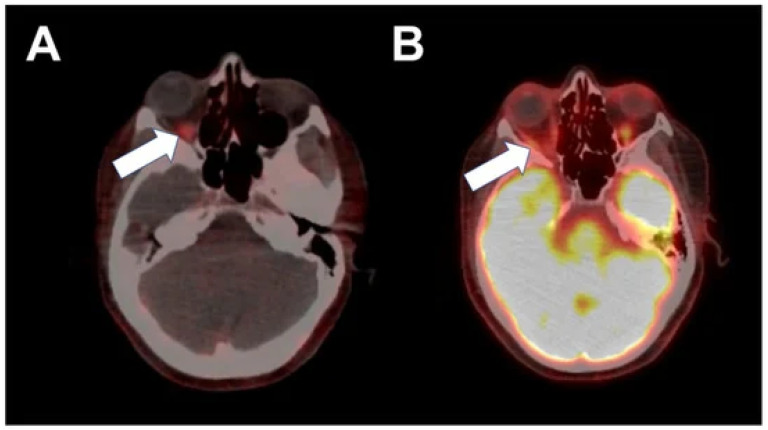
Abnormal intense [^18^F]FES activity in the right posterior orbit ((**A**), arrow) corresponding to the known right oculomotor nerve, right medial rectus, and superior aspect of the right inferior rectus. Normal physiologic [^18^F]FDG activity in the right extraocular muscles ((**B**), arrow) [31].

Contemporary research in the field of breast cancer has underscored a rising occurrence of orbital metastases in individuals diagnosed with metastatic breast cancer (MBC) [31]. These metastatic growths frequently affect the extraocular muscles and orbital adipose tissue, resulting in symptoms like ocular dysmotility, double vision, eye protrusion, reduced visual acuity, discomfort, and eyelid drooping [31]. The diagnostic process for orbital metastases is complex, often necessitating invasive methods such as surgical biopsies or fine-needle aspiration, which are associated with potential complications including hemorrhage, loss of vision, and infection. Predominantly, over 60% of breast cancers exhibit estrogen receptor positivity (ER+), indicating estrogen’s crucial role in cellular proliferation and growth. In this scenario, the innovative radiotracer ^18^F-fluoroestradiol ([^18^F]FES) has gained prominence as an effective imaging tool for ER+ MBC [31]. This is particularly true in regions of naturally high glucose uptake where conventional ^18^F-fluorodeoxyglucose ([^18^F]FDG) PET scans may be less effective. The clinical adoption of [^18^F]FES is bolstered by numerous studies, showcasing its efficacy in identifying ER+ metastases, especially in low-grade malignancies and in locations with elevated baseline physiologic FDG uptake. For an illustrative example of the intense [^18^F]FES activity in the right posterior orbit, indicative of ER+ metastases, see Figure 1, which demonstrates the clinical relevance of this novel radiotracer.

### 1.2. The MTHFD2 Protein

Despite the progress made so far, achieving accuracy and effectiveness when targeting cancer remains a challenging task [32]. One of the obstacles is the need for specificity in targeting specific molecular pathways or biomarkers associated with tumor formation [33]. The increased expression or disruption of proteins within cancer cells, such as metabolic enzymes or signaling molecules, provides an opportunity to personalize the design of radiopharmaceuticals [34,35]. Among these proteins, methylenetetrahydrofolate dehydrogenase 2 (MTHFD2), which plays a role in the metabolic pathway, has attracted significant attention due to its heightened presence across different types of solid tumors [36,37].

MTHFD2 is an enzyme involved in one-carbon metabolism within the mitochondria and is upregulated in various types of solid tumors [36,38], such as lung adenocarcinoma [36], hepatocellular carcinoma [39], renal cell carcinoma [40], colorectal cancer [41], and breast cancer [42]. The overexpression of MTHFD2 not only supports cancer cell migration and invasion but also indicates poor clinical outcomes [39,43]. Recent research has identified MTHFD2 as having roles in redox defense, epigenetic modification, RNA translation, and DNA repair [44]. The knockdown of MTHFD2 in colorectal cancer reduces NADPH production and makes cancer cells more vulnerable to oxidative stress [45]. In renal cell carcinoma, MTHFD2 promotes the methylation of HIF-2α mRNA through metabolic reprogramming [40]. Additionally, MTHFD2 is found to play a role in RNA metabolism and translation by interacting with nuclear proteins in cancer cells [46].

The recognition of MTHFD2’s pervasive presence and multifaceted roles across a spectrum of cancers underscores the quest for targeted interventions tailored to combat its influence [47]. As part of this endeavor, anastrozole and epirubicin, which are known compounds with distinct mechanisms of action in solid tumor treatment, have garnered attention for their potential efficacy in addressing MTHFD2-driven tumors [14]. Anastrozole, an aromatase inhibitor primarily used in hormone receptor-positive breast cancer, exerts its effect by suppressing estrogen synthesis [48]. On the other hand, epirubicin, a chemotherapeutic agent belonging to the anthracycline class, demonstrates efficacy against various cancer types by inducing DNA damage [49]. The distinct mechanisms of action exhibited by anastrozole and epirubicin, which are known for their efficacy in specific cancer treatments, suggest a potential dual functionality [50,51]. Apart from their established roles in tumor treatment, these compounds possess inherent attributes that could facilitate their utilization as effective carriers for delivering radiotracers to solid tumors characterized by heightened MTHFD2 expression [14]. This dual functionality underscores their candidacy as potential targeting agents, emphasizing their versatility in not only combating tumor cells but also in aiding targeted delivery for diagnostic radiopharmaceuticals in MTHFD2-driven malignancies.

### 1.3. Radiopharmaceutical Therapy

**Figure 2:**
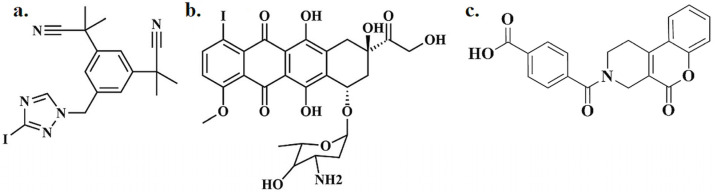
Radioiodinated anastrozole ([^125^I]anastrozole) (**a**), radioiodinated epirubicin ([^125^I]epirubicin) (**b**), and DS44960156 (**c**).

Radiopharmaceuticals bearing radioactive iodine on an sp3 carbon atom are uncommon, but some compounds have been shown to be effective in treating solid tumors [52]. In the past, molecules such as iododoxorubicin have represented significant advancements, showcasing the potential of radioiodinated derivatives in oncology [53]. Studies have explored the biological evaluation of iododoxorubicin, highlighting its increased tumor uptake and favorable pharmacokinetic profile compared to its non-radioiodinated counterparts [53,54,55]. Radioiodinated anastrozole ([^125^I]anastrozole) and radioiodinated epirubicin ([^125^I]epirubicin) (Figure 2) [14], for example, have been studied as potential targeting radiopharmaceuticals for solid tumor imaging. This study describes the preparation of [^125^I]anastrozole and [^125^I]epirubicin and their biological evaluation as potential solid tumor imaging agents. The radiochemical yields for [^125^I]anastrozole and [^125^I]epirubicin were maximized to 92.9 ± 0.1 and 98.8 ± 0.1%, respectively, and they showed in vitro stability up to 4 and 24 h, respectively. The preclinical evaluation and biodistribution in mice bearing solid tumors showed high retention and biological accumulation in solid tumor cells and high T/NT ratios equal to 4.7 ± 0.1 and 5.2 ± 0.1 at 2 and 1 h post-injection, respectively. The data described before could recommend [^125^I]anastrozole and [^125^I]epirubicin as potential targeting radiopharmaceuticals for solid tumor imaging [14].

### 1.4. The Aim of the Study

While [^125^I]anastrozole and [^125^I]epirubicin show promise in targeting solid tumor imaging, elucidating their precise interactions with enzymes remains a challenge. Traditional methods used to determine the effectiveness of enzyme inhibition can be both complex and costly. Herein lies the value of computational techniques, such as molecular docking and molecular dynamics simulations. These methods provide cost-effective and efficient ways to examine the interaction dynamics between these radioiodinated compounds and the MTHFD2 enzyme. Our primary objective is to utilize these computational strategies to assess the binding affinity and structural integrity of the compound–enzyme complex during simulations. This investigation seeks to elucidate the binding mechanisms of radioiodinated compounds with the MTHFD2 enzyme and hopes to demonstrate the potential of computational methodologies in accelerating the development of targeted imaging agents for solid tumors implicated with MTHFD2.

## 2. Materials and Methods

### 2.1. Molecular Docking Preparation

In this investigation, our attention was directed towards the MTHFD2 enzyme, which was studied in conjunction with a potent isoenzyme inhibitor, DS44960156 [56]. MTHFD2 was chosen for its prevalent role in several cancer cell lines and the significant implications of its inhibition on cellular growth and survival [57,58,59,60]. We obtained the crystallographic data of MTHFD2 in a complex with DS44960156 (PDB ID: 6JIB) from the Protein Data Bank (PDB) [61] with the from 15 December 2023. To establish a baseline for comparison, DS44960156, which was co-crystalized within the enzyme complex, was used as a reference in this study.

For the molecular docking analysis, the three-dimensional structure of the enzyme target underwent a series of preprocessing steps so that it could be prepared. Also, the extraneous non-crucial water molecules and heteroatoms were removed by utilizing the Biovia Discovery Studio Visualizer [62], yielding a refined structure in PDB format. The YASARA web server was instrumental in adding any missing amino acids to the enzyme’s structure [63,64,65]. At a pH of 7.4, the ionization states of amino acids capable of titration were calculated using the H^++^ web server [66]. Subsequently, AutoDock Tools version 1.5.6 played a crucial role in appending polar hydrogen atoms and Kollman charges to alter the structure into PDBQT format for further processing [67].

This study also emphasized the preparation and optimization of ligands for molecular docking simulations, aiming to enhance the accuracy and reliability of the findings. The ChemDraw JS web server [64] was utilized to draw [^125^I]anastrozole and [^125^I]epirubicin based on structures proposed in previous research [14], and they were stored in Structural Data File format. The ligands’ stability was confirmed by subjecting them to an energy minimization procedure. This was accomplished by employing the Universal Force Field combined with a Conjugate Gradient optimization algorithm, as referenced in [68]. The process, executed over a course of a thousand steps, was carried out using the Open Babel software version 2.3 [69]. Subsequently, the resulting structures were stored in PDB format. During this phase, Gasteiger charges were allocated utilizing AutoDock Tools version 1.5.6. Then, the structures were formatted into PDBQT files, priming them for subsequent docking simulations.

To study the interaction between the ligand and the MTHFD2 active site, molecular docking was performed using AutoDock 4.2. This involved employing a Lamarckian genetic algorithm for optimization purposes [70]. We examined various molecular configurations within the binding site, allowing for ligand flexibility while keeping the macromolecule rigid. The grid box for the enzyme was set at dimensions of 40 × 40 × 40 along the X, Y, and Z axes, with the central grid point coordinates fixed at −62.96, 30.15, and −26.28. The docking process included 100 runs with a maximum of 25,000,000 evaluations, and default parameters were used to ensure consistency across calculations.

### 2.2. Molecular Dynamics Simulations

This study was principally focused on evaluating the binding behavior and stability of [^125^I]anastrozole and [^125^I]epirubicin within the active sites of the MTHFD2 enzyme. These assessments were conducted through molecular dynamics simulations. Our simulations commenced with the human crystal structure of MTHFD2 in a complex with the DS44960156 inhibitor. For the execution of the molecular dynamics simulations, we used the GROMACS software package, version 2016.3, and applied the Gromos96 54a7 force field [71]. Spanning 200 ns, these simulations offered an in-depth evaluation of the ligands’ interactions and stability within the active sites of the enzyme.

Prior to starting the simulation, topology files for both the ligand and protein were created using specific tools. The GROMACS tool ‘pdb2gmx’ was used for the ligand, and the protein’s topology was generated via the PRODRG server, which was accessed on 23 December 2023 at http://davapc1.bioch.dundee.ac.uk/cgi-bin/prodrg. Following this, we immersed the systems in a solvent by employing the Transferable Intermolecular Potential 3 Points (TIP3P) water model and added counterions to balance the overall charge. The preparation phase was concluded by minimizing the potential energy of the systems. This was achieved using the steepest descent integrator for 50,000 steps, with each step sized at 0.01 in energy units. After this, the systems were initially equilibrated in the NVT ensemble for 100 ps at 310 K using the v-rescale method, and then in the NPT ensemble for another 100 picoseconds at 1.0 bar, by employing the Berendsen pressure coupling method [72,73].

After completing the equilibration of the thermal as well as pressure conditions, MD simulations were conducted for 200 ns each at 1 bar pressure and a 310 K temperature. A cut-off distance of 1.0 nm was utilized for short-range non-bonded interactions, and the Particle Mesh Ewald (PME) method was usesd for long-range electrostatics [74]. The LINCS algorithm was used to constrain bonds involving hydrogen atoms [75]. Throughout the simulations, a 2 fs timestep was used, and coordinates were reset every 5000 steps (equivalent to 10 ps) during the processing of the molecular dynamics data. We applied this method to ensure accuracy and reliability in order to obtain dependable outcomes while maintaining simulation integrity. To evaluate the robustness and stability of the interactions between the compounds and the enzyme, a range of tools and techniques including RMSD, RMSF, RG, and H-bond analysis were utilized in our analysis of MD trajectories.

### 2.3. MM-PBSA Calculation

In this study, we utilized the MM-PBSA method, which is widely acknowledged for its effectiveness in calculating binding free energies from molecular dynamics simulations. We focused on the equilibrium phase of these simulations to evaluate the binding free energies involving [^125^I]anastrozole, [^125^I]epirubicin, and DS44960156 in interactions with the MTHFD2 enzyme. During the simulations, we systematically captured snapshots every 100 ps in a timeframe extending from 180 to 200 ns. This was achieved using the g_mmpbsa tool integrated within the Gromacs software suite (version 2016.3) [76,77]. To determine the binding free energy of the ligand–protein complex in the solvent environment, the sum of the individual total energies of the protein (*G*_protein_) and ligand (*G*_ligand_) when they were in the solvent separately were subtracted from the overall free energy of the combined protein–ligand complex (*G*_complex_). This calculation employs the following formula [78]:

Δ*G*_bind_ = [*G*_complex_ − *G*_protein_ + *G*_ligand_] (1)

The free energy for each distinct state, encompassing the complex, protein, and ligand, is estimated by summing the average molecular mechanics potential energy in a vacuum (*E*_MM_) and the solvation free energy (*G*_solvation_) [77]:

*G*x = *E*_MM_ + *G*_solvation_ (2)

The calculation of a molecule’s potential energy in a vacuum involves summing the energies arising from bonded (*E*_bonded_) and non-bonded (*E*_non-bonded_) interactions. Specifically, *E*_bonded_ comprises the energy contributions from bonding, angular, dihedral, and improper interactions. On the other hand, Enon-bonded includes the sum of electrostatic (*E*_elec_) and Van der Waals (*E*_vdw_) interactions [77]:

*E*_MM_ = *E*_bonded_ + *E*_non_bonded_ = *E*_bonded_ + [*E*_vdw_ + *E*_elec_] (3)

The solvation free energy is further divided into the non-polar free energy of solvation (*G*_non-polar_) and the electrostatic solvation free energy (*G*_polar_) [77]:

*G*_solvation_ = *G*_polar_ + *G*_non-polar_ (4)

To compute the electrostatic solvation free energy (*G*_polar_), the Poisson–Boltzmann equation was applied [79]. Additionally, the solvent-accessible surface area (SASA) method was used to estimate the non-polar solvation free energy (*G*_non-polar_), which was determined based on the following equation:

*G*_non-polar_ = *γ*_SASA_ + *b* (5)

In this study, two distinct sets of coefficients, *γ* and *b*, were employed to calculate the solvation free energy, as outlined in Equation (5). For simulations conducted in units of kJ/mol, the coefficients selected were *γ* = 0.02267 kJ/mol·Å^2^ and *b* = 3.849 kJ/mol. The choice of these coefficient sets is contingent on the unit system adopted for the simulation. It is important to note that, in these calculations, Δ*E*_bonded_ was presumed to be zero [80]. These binding free energy calculations are significant as they offer valuable insights into the energy dynamics governing the interactions between protein and ligand molecules and can be instrumental in identifying potential hit compounds.

## 3. Results

### 3.1. Molecular Docking

**Figure 3:**
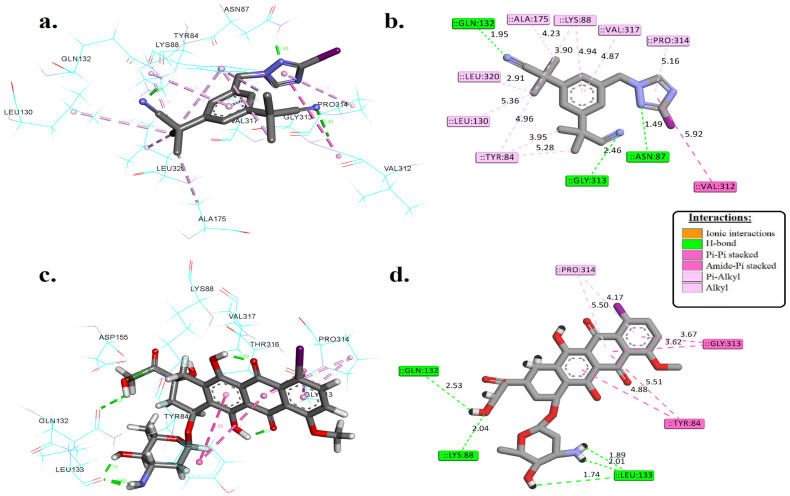
The 3D and 2D interactions of [^125^I]anastrozole (**a**,**b**) and [^125^I]epirubicin (**c**,**d**) within the active binding site of the human MTHFD2 enzyme (PDB ID: 6JIB). These models were generated using the Discovery Studio visualizer.

In this study, we conducted molecular docking simulations involving [^125^I]anastrozole, [^125^I]epirubicin, and a co-crystallized ligand, DS44960156—a selective Methylenetetrahydrofolate dehydrogenase 2 (MTHFD2) inhibitor with an IC50 value of 1.6 μM [56]. This compound was used as a control to compare the molecular interactions of the radioiodinated compounds with the MTHFD2 enzyme. The findings from these simulations are summarized in Figure 3 and detailed in Table 1.

### 3.2. Molecular Dynamics Simulation

**Figure 4:**
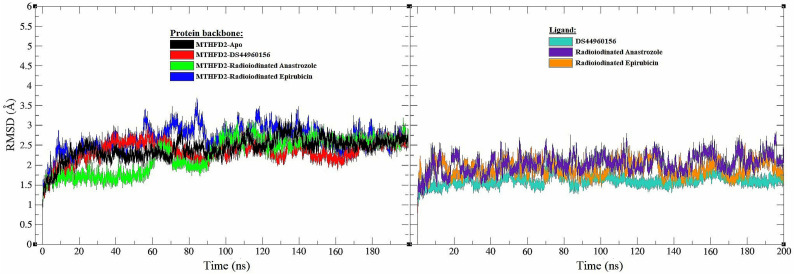
The root mean square deviation (RMSD) plots of the MTHFD2 enzyme and ligand backbone atoms for the selected systems: MTHFD2-Apo (black), MTHFD2-DS44960156 (red and turquoise), MTHFD2-[^125^I]anastrozole (green and indigo), and MTHFD2-[^125^I]epirubicin (blue and orange).

**Figure 5:**
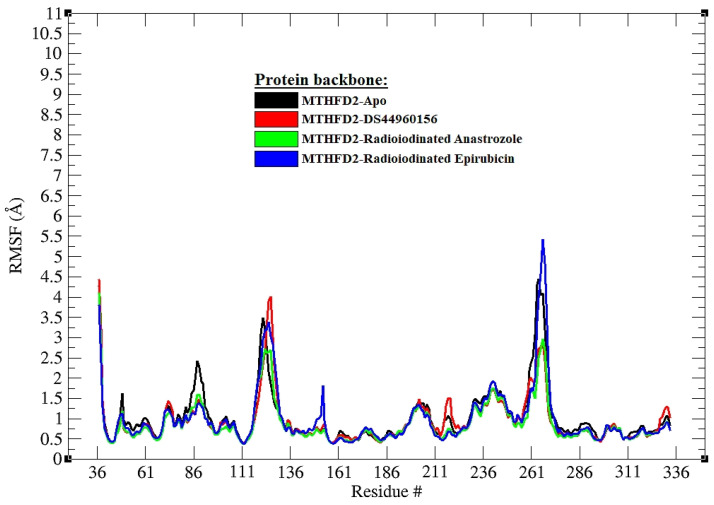
The root mean square fluctuation (RMSF) diagrams of the backbone atoms in MTHFD2 during a 200 ns molecular dynamics simulation for all systems are depicted. The RMSF values reflect the remaining atomic fluctuations of each protein residue in their interaction with ligands throughout this progression path.

**Figure 6:**
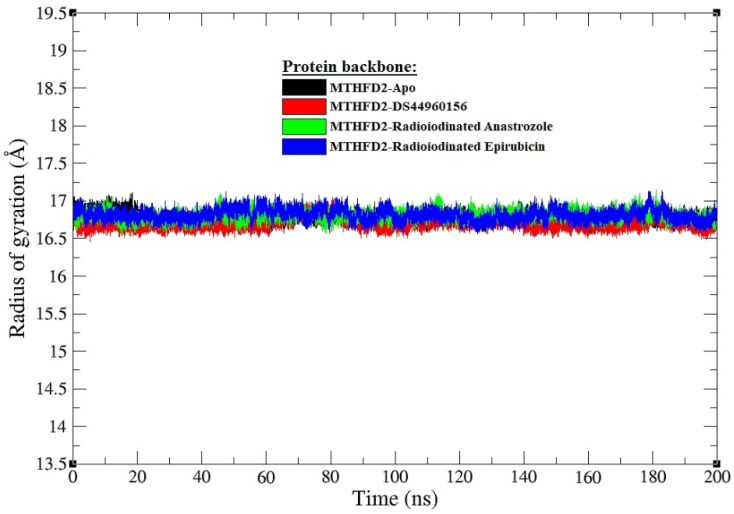
Radius of gyration (RG) graphs for all systems’ MTHFD2 backbone atoms at MD interval timeframes (0–200 ns); radii depicted in apo form and in post-bound interactions of DS44960156, [^125^I]anastrozole, and [^125^I]epirubicin with MTHFD2 enzyme are illustrated in black, red, green, and blue, respectively.

**Figure 7:**
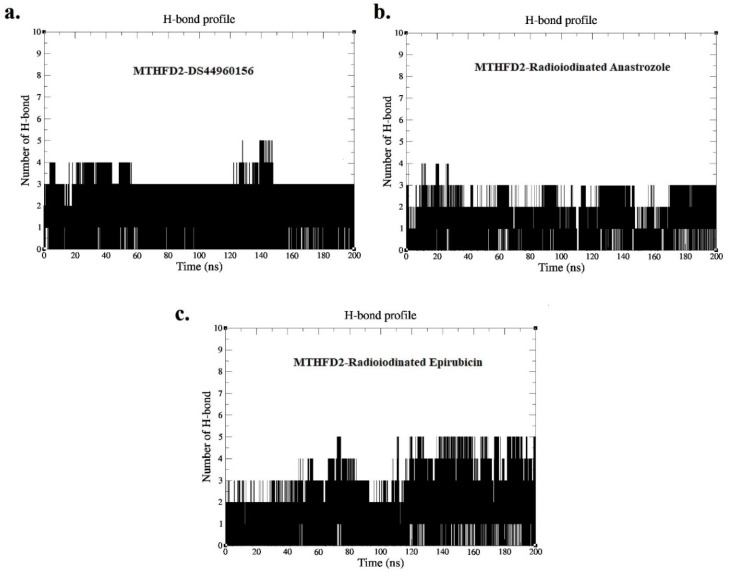
Hydrogen bond profile acquired from MD simulation over time period of 0–200 ns for (**a**) MTHFD2-DS44960156, (**b**) MTHFD2-[^125^I]anastrozole, and (**c**) MTHFD2-[^125^I]epirubicin.

A 200 ns molecular dynamics simulation (MD) was performed using GROMACS 2016 to evaluate the binding of [^125^I]anastrozole and [^125^I]epirubicin to MTHFD2’s active site. This simulation was compared with the results of the co-crystallized ligand (DS44960156). Various analyses were carried out during the simulation, including root mean square deviation (RMSD), root mean square fluctuation (RMSF), radius of gyration (RG), hydrogen bond interactions (H-bond), and molecular mechanics Poisson–Boltzmann surface area (MM-PBSA) of the enzyme in complex with the ligand’s backbone atoms. The results of these analyses are presented in Figure 4, Figure 5, Figure 6 and Figure 7, as well as in Table 2.

## 4. Discussion

### 4.1. Molecular Docking

Molecular docking is a vital computational method that predicts how a molecule, typically a small ligand, interacts with a protein to form a stable complex, revealing insights into binding orientations and affinities [81]. This technique is instrumental in drug design, allowing researchers to explore potential inhibitory effects on target enzymes [82]. Redocking the co-crystalized ligand validates the docking parameters by ensuring the computational model can recapitulate the experimentally determined ligand orientation into the active binding site of the enzyme [83].

The redocking of the co-crystalized ligand (DS44960156) within the MTHFD2 enzyme’s active site exhibits remarkable fidelity to the original conformation, as reflected by an RMSD value of ~0.84 Å (Figure S1). This close alignment in panel (a) between the original ligand (depicted in green) and the re-docked ligand (in orange) underscores the robustness of the docking process used. The detailed 2D interaction analysis in panels (b) and (c) further highlights the specific amino acids involved in the interaction with the ligand, such as the hydrogen bonds with amino acid residues such as GLY310 and ASN87, the ionic interaction with ARG43, and the π–alkyl interaction with PRO314, among others. These interactions are pivotal for the ligand’s binding affinity, and their retention in the re-docked structure validates the accuracy of the docking parameters. Hence, Figure S1 not only confirms the reliability of the docking procedure but also supports the integrity of the subsequent computational exploration of [^125^I]anastrozole and [^125^I]epirubicin as targeting agents for MTHFD2-driven tumors.

Figure 3 and Table 1 present a detailed comparative analysis of the binding free energies and molecular interactions of [^125^I]anastrozole and [^125^I]epirubicin within the active site of the MTHFD2 enzyme. Notably, [^125^I]epirubicin demonstrates a more negative binding free energy (∆*G*_bind_) of −41.25 kJ/mol, in contrast to the binding free energies of −39.07 kJ/mol for [^125^I]anastrozole and −38.53 kJ/mol for the co-crystallized ligand, indicating a potentially enhanced affinity towards the MTHFD2 enzyme.

When exploring the interaction profile, it was found that [^125^I]anastrozole forms critical hydrogen bonds with ASN87, GLN132, and GLY313 at distances of 1.49, 1.95, and 2.46 Å, respectively. These interactions are pivotal for its binding efficacy. Conversely, [^125^I]epirubicin establishes hydrogen bonds with LYS88 (2.04 Å) and GLN132 (2.53 Å) and engages in multiple interactions with LEU133 (1.74, 1.89, and 2.01 Å), suggesting a robust binding network, likely contributing to its higher binding affinity.

Remarkably, while ionic interactions are absent in the radioiodinated compounds, the co-crystallized ligand forms an ionic bond with ARG43, indicating a distinct binding modality. Additionally, hydrophobic interactions involving residues such as TYR84, LYS88, and PRO314 for both radioiodinated compounds, supplemented by additional contacts in [^125^I]anastrozole with LEU130, ALA175, and others, highlight the critical role of hydrophobic effects in stabilizing these interactions.

Given the promising binding energies and interaction profiles, these radioiodinated compounds merit further exploration. Molecular dynamics simulations would offer deeper insights into the stability and dynamics of these interactions over time, providing a more comprehensive understanding of the binding processes.

### 4.2. Molecular Dynamics (MD) Simulation

MD simulations are indispensable in elucidating the time-dependent behaviors of biological molecules. They offer invaluable insights into the conformational stability and dynamic interactions inherent within protein–ligand complexes. In Figure 4, we observe the RMSD of both protein and ligand backbones throughout a 200 ns MD simulation, which sheds light on the structural integrity of the MTHFD2 enzyme when complexed with various ligands. The apo form of MTHFD2, represented by a black trace in the graph, establishes a baseline RMSD, facilitating the assessment of how ligand binding influences the protein’s structural stability. The protein backbone complexed with the co-crystallized ligand, depicted in red, demonstrates an average RMSD, which is indicative of a stable interaction over the simulation period. Similarly, the complexes with [^125^I]anastrozole (green) and [^125^I]epirubicin (blue) exhibit stable RMSD trajectories, with average values within a comparable range. This suggests that the process of radioiodination does not adversely affect the structural integrity of the enzyme–ligand complex.

When focusing on the ligands, it can be seen that the backbone RMSD of the co-crystallized ligand (turquoise), alongside those of [^125^I]anastrozole (indigo) and [^125^I]epirubicin (orange), reveal that the radioiodinated ligands maintain a consistent binding conformation. The average RMSD values of these ligands are closely aligned with those of the control, indicating conformational stability. This is a key indicator of a robust interaction within the active site and bodes well for the potential efficacy of these compounds as radiopharmaceuticals. The preservation of structural integrity in the MTHFD2 enzyme upon binding with [^125^I]anastrozole and [^125^I]epirubicin is indeed promising. Yet, it is imperative to delve deeper into these observations through a root mean square fluctuation analysis. This will allow us to evaluate the flexibility of individual amino acid residues in the enzyme. Such a nuanced analysis will reveal whether these ligands induce any localized changes in flexibility that could potentially impact enzyme functionality. The correlation between the stability observed in the RMSD analyses and the flexibility potentially unveiled by the RMSF studies will furnish a more holistic understanding of the effects that these novel radiopharmaceutical candidates exert on the MTHFD2 enzyme. This comprehensive approach is crucial in advancing our understanding of these compounds and their potential therapeutic applications.

Figure 5 provides a detailed RMSF analysis for the backbone atoms of the MTHFD2 enzyme across various ligand-bound states and the apo form during a 200 ns molecular dynamics simulation. The RMSF values are crucial for understanding the flexibility of each residue when influenced by different ligands or in the unbound state. In the graph, it can be observed that the apo form of MTHFD2 (black) serves as a baseline, exhibiting inherent flexibility across the protein structure. The ligand-bound states modify this inherent flexibility to varying degrees. In the comparison, the MTHFD2 complex with [^125^I]anastrozole (green) and [^125^I]epirubicin (blue) shows a generally similar pattern of fluctuations to those of the co-crystallized ligand (red) and the apo form, suggesting that the binding of these compounds does not drastically alter the protein’s dynamic behavior. However, a careful inspection of the graph might reveal specific residues where the RMSF peaks differ significantly from the apo form. These particular residues, which could show higher or lower flexibility upon ligand binding, may be crucial for the protein’s functional conformation and could be investigated further for their role in ligand specificity and binding affinity.

The RG is a pivotal metric for assessing the overall compactness and stability of protein structures in molecular dynamics simulations. Figure 6 delineates the Rg for the MTHFD2 enzyme across various states: the unbound apo form and complexes with different ligands. The apo MTHFD2 form displays an average Rg of approximately 16.85 Å, indicating a certain degree of compactness intrinsic to the unbound protein. Comparatively, the complex with the co-crystallized ligand exhibits a slightly more compact average Rg of about 16.75 Å. This subtle reduction in the Rg value suggests that the binding of the co-crystallized ligand may induce a marginal increase in the compactness of the enzyme, which is consistent with the formation of a stable protein–ligand complex. In the context of the radioiodinated compounds, the complex with [^125^I]anastrozole shows an average Rg of approximately 16.90 Å, which is slightly higher than both the apo form and the co-crystallized ligand complex. This suggests a minimal expansion in the overall structure, which could be attributed to the specific interactions and possibly the steric effects introduced by the [^125^I]anastrozole. Nevertheless, the deviation is minor and falls within a range that does not indicate significant unfolding or destabilization. On the other hand, the complex with [^125^I]epirubicin has an average Rg of about 16.84 Å, closely mirroring the compactness of the apo enzyme. This resemblance implies that the [^125^I]epirubicin maintains the protein’s structural integrity to a degree very similar to that of the unbound state.

Figure 7 illustrates the hydrogen bond profiles of the MTHFD2 enzyme in a complex with its co-crystallized ligand (DS44960156), [^125^I]anastrozole, and [^125^I]epirubicin over the course of a 200 ns molecular dynamics simulation. The average number of consistent hydrogen bonds formed between the co-crystallized ligand and the MTHFD2 active site is three, which is indicative of a stable interaction throughout the simulation period. This baseline of hydrogen bonding is essential for understanding the comparative binding profiles of the radioiodinated compounds. For the complex with [^125^I]anastrozole, the average number of consistent hydrogen bonds observed is two. Although this is slightly lower than the control, it still suggests a stable interaction, which could be sufficient for therapeutic efficacy depending on the specific nature and strength of these bonds. The consistent formation of two hydrogen bonds over time implies that, while the modification may slightly reduce the number of interactions compared to the native ligand, the binding mode remains robust. In the case of [^125^I]epirubicin, the H-bond profile is consistent with the control, maintaining an average of three hydrogen bonds with the MTHFD2 active site. This similarity in hydrogen bonding behavior suggests that [^125^I]epirubicin maintains a level of interaction comparable to the native ligand, which is promising for its functional activity. The analysis of these hydrogen bond profiles is crucial because it relates directly to the stability and specificity of ligand binding. A consistent hydrogen bond number throughout the simulation suggests that the ligands are capable of maintaining a steady interaction with the enzyme, which is a desirable trait for the development of radiopharmaceuticals that may require prolonged or targeted interaction with the enzyme.

The stability of these average RG values, along with the findings from the RMSD and RMSF analyses, suggests that both [^125^I]anastrozole and [^125^I]epirubicin form stable complexes with the MTHFD2 enzyme. Their ability to maintain the enzyme’s structural compactness, coupled with favorable hydrogen bonding interactions as suggested by H-bond studies, presents a promising profile for these compounds as potential radiopharmaceuticals.

To further substantiate these findings, the molecular mechanics Poisson–Boltzmann surface area (MM-PBSA) method could be employed. MM-PBSA is a post-simulation analysis that estimates the free energy of binding between a protein and a ligand by taking into account the contributions from molecular mechanics, solvation effects, and entropy. Applying MM-PBSA to these complexes could provide a more detailed assessment of the binding affinity and could help to clarify the implications of the observed differences in the hydrogen bonding profiles. Such an assessment would be particularly useful in validating the potential of [^125^I]anastrozole and [^125^I]epirubicin to be used as targeting agents by providing a more comprehensive understanding of their interaction strength with the MTHFD2 enzyme. Table 2 presents the MM-PBSA-calculated binding energies for the interactions of the co-crystallized ligand (DS44960156), [^125^I]anastrozole, and [^125^I]epirubicin with the MTHFD2 enzyme. The MM-PBSA method provides a comprehensive assessment of the binding affinity by considering various energetic components, which are crucial for understanding the strength and nature of ligand–enzyme interactions. For the MTHFD2-DS44960156 control complex, the overall ΔG binding is quite favorable at −50.57 ± 0.16 kJ/mol, which is driven by strong electrostatic and Van der Waals interactions, which are −34.15 ± 0.18 kJ/mol and −17.88 ± 0.19 kJ/mol, respectively. These interactions are partially offset by the polar solvation energy (18.41 ± 0.11 kJ/mol), which is a cost of desolvating the ligand and the active site for binding to occur. The non-polar solvation component, which often corresponds to the hydrophobic effect, further stabilizes the complex with a value of −16.95 ± 0.13 kJ/mol. In comparison, the MTHFD2-[^125^I]anastrozole complex shows a Δ*G* _bind_ of −48.49 ± 0.11 kJ/mol. The major contributors to this binding energy are the Van der Waals component (−20.88 ± 0.19 kJ/mol), which is higher than that of the control, suggesting a favorable hydrophobic interaction, and the electrostatic interactions (−30.15 ± 0.21 kJ/mol), which are slightly less favorable than in the control. The polar and non-polar solvation energies are comparable to the control, indicating similar desolvation costs and hydrophobic contributions to binding. The MTHFD2-[^125^I]epirubicin complex has a Δ*G*_bind_ of −49.53 ± 0.12 kJ/mol, indicating a binding affinity that is intermediate between the control and [^125^I]anastrozole. The electrostatic contribution (−33.41 ± 0.17 kJ/mol) is closer to the control, while the Van der Waals interactions (−17.61 ± 0.20 kJ/mol) and solvation energies are comparable to the other two systems. These findings suggest that both [^125^I]anastrozole and [^125^I]epirubicin have the potential to inhibit the MTHFD2 enzyme, with binding affinities that are in the same range as the co-crystallized ligand, albeit slightly lower. The major contributors to binding energy, notably the Van der Waals interactions for [^125^I]anastrozole and the electrostatic interactions for [^125^I]epirubicin, play significant roles in the binding of these ligands.

While the MM-PBSA scores provide theoretical insights into the binding affinities of these compounds, it is crucial to recognize that in silico predictions need to be validated through experimental studies. When developing radioiodinated radiopharmaceuticals, such as [^125^I]anastrozole and [^125^I]epirubicin, one must consider the phenomenon of in vitro deiodination [84]. This process can significantly impact the stability, biodistribution, and, ultimately, the therapeutic efficacy of these agents [84]. Deiodination in the body leads to the release of free iodine, which can alter the intended targeting properties of the compounds and potentially increase the dose to non-target tissues [85]. While radioiodinated pharmaceuticals are well established for their utility in diagnostics and therapy, addressing the challenge of deiodination is crucial to maximize their clinical effectiveness [86]. Strategies to mitigate in vivo deiodination include the chemical modification of the compound to reduce susceptibility to enzymatic cleavage and the use of stabilizing agents. Further investigation into the mechanisms of deiodination and the development of strategies to counteract this process will be essential for the advancement of [^125^I]anastrozole and [^125^I]epirubicin as targeted radiopharmaceuticals for MTHFD2-driven neoplasms. Also, in vitro assays to evaluate the inhibitory activity of these compounds against MTHFD2, followed by in vivo studies to assess their efficacy and safety profiles, are essential to support the computational findings. Such multi-level validation would be instrumental in confirming the therapeutic potential of [^125^I]anastrozole and [^125^I]epirubicin as inhibitors of the MTHFD2 enzyme.

## 5. Conclusions

This study examines the potential of [^125^I]anastrozole and [^125^I]epirubicin to target MTHFD2-driven tumors using comprehensive molecular docking and dynamics simulations. The molecular docking scores reveal that [^125^I]epirubicin has a superior binding affinity to MTHFD2, which is underscored by a higher binding free energy compared to those of [^125^I]anastrozole and the control ligand. The structural integrity and interaction dynamics of the enzyme–ligand complexes, assessed through RMSD, RMSF, and RG metrics, suggest minimal structural disruption and stable binding over time. Notably, the hydrogen bond analysis and MM-PBSA calculations further confirm the thermodynamic favorability of these interactions, indicating the compounds’ potential to be used as precise imaging and therapeutic agents in oncology.

This investigation emphasizes the pivotal role of computational tools in predicting the efficacy of novel radiopharmaceuticals, setting a foundation for their subsequent empirical validation. While the findings suggest that [^125^I]anastrozole and [^125^I]epirubicin are promising MTHFD2 inhibitors, it is crucial to conduct in vitro and in vivo assays to fully ascertain their therapeutic utility and safety profiles. Hence, our research marks an important step toward enhancing precision oncology, with future studies poised to expand on this preliminary groundwork.

## Figures and Tables

**Table 1:** The free binding energy values (Δ*G*_bind_ (kJ/mol)) and molecular interactions analysis of radioiodinated anastrozole ([^125^I]anastrozole), radioiodinated epirubicin ([^125^I]epirubicin), and the co-crystallized ligand (DS44960156) within the active binding site of the human MTHFD2 enzyme (PDB ID: 6JIB) using AutoDock 4.2.

Compounds	Δ*G*_bind_ (kJ/mol)	Hydrogen Bond Interactions		Ionic Interaction	Hydrophobic Interaction
		Residues	Distances (Å)		
[^125^I]anastrozole	−39.07	ASN87, GLN132, and GLY313	1.49, 1.95, and 2.46	-------	TYR84, LYS88, LEU130, ALA175, VAL312, PRO314, VAL317, and LEU320
[^125^I]epirubicin	−41.25	LYS88, GLN132, LEU133, LEU133, and LEU133	2.04, 2.53, 1.74, 1.89, and 2.01	-------	TYR84, GLY313, and PRO314
Co-crystalized ligand	−38.53	ASN87, LYS88, GLN132, and GLY310	1.73, 1.48, 1.95, and 2.05	ARG43	TYR84, GLY313, and PRO314

**Table 2:** MM-PBSA binding energies (Δ*G*_bind_) of [^125^I]anastrozole, [^125^I]epirubicin, and DS44960156 (control) at active site of MTHFD2 (PDB ID: 6JIB) enzyme. Energy units are expressed in kJ/mol.

System	Δ*G*_Binding_ (kJ/mol)	Electrostatic (kJ/mol)	Van der Waal (kJ/mol)	Polar Salvation (kJ/mol)	Non-Polar Salvation (kJ/mol)
**MTHFD2-DS44960156**	−50.57 ± 0.16	−34.15 ± 0.18	−17.88 ± 0.19	18.41 ± 0.11	−16.95 ± 0.13
**MTHFD2**-[^125^I]anastrozole	−48.49 ± 0.11	−30.15 ± 0.21	−20.88 ± 0.19	18.75 ± 0.14	−16.21 ± 0.14
**MTHFD2-[**^125^I]Epirubicin	−49.53 ± 0.12	−33.41 ± 0.17	−17.61 ± 0.20	17.92 ± 0.12	−16.43 ± 0.14

## Data Availability

Data is contained within the article and Supplementary Materials.

## Supplementary Materials

The following supporting information can be downloaded at https://www.mdpi.com/article/10.3390/pharmaceutics16050616/s1, Figure S1. (a) The superimposition and (b) 2D interaction analysis of the co-crystallized ligand (DS44960156) (highlighted in green for carbon (C), red for oxygen (O), and navy for nitrogen (N)). (c) The re-docked ligand (highlighted in orange for carbon (C), red for oxygen (O), and navy for nitrogen (N)) within the crystal structure of the human MTHFD2 enzyme (PDB ID: 6JIB).
