# Supplementary material for: Radiological and Molecular Analysis of Radioiodinated Anastrozole and Epirubicin as Innovative Radiopharmaceuticals Targeting Methylenetetrahydrofolate Dehydrogenase 2 in Solid Tumors
Source: Pharmaceutics. 2024 May 3;16(5):616. doi: 10.3390/pharmaceutics16050616 (PMC11126143; doi:10.3390/pharmaceutics16050616)
Supplement: Supplementary file 1 [file pharmaceutics-16-00616-s001.zip › pharmaceutics-2902981-supplementary.pdf]

Supplementary Materials

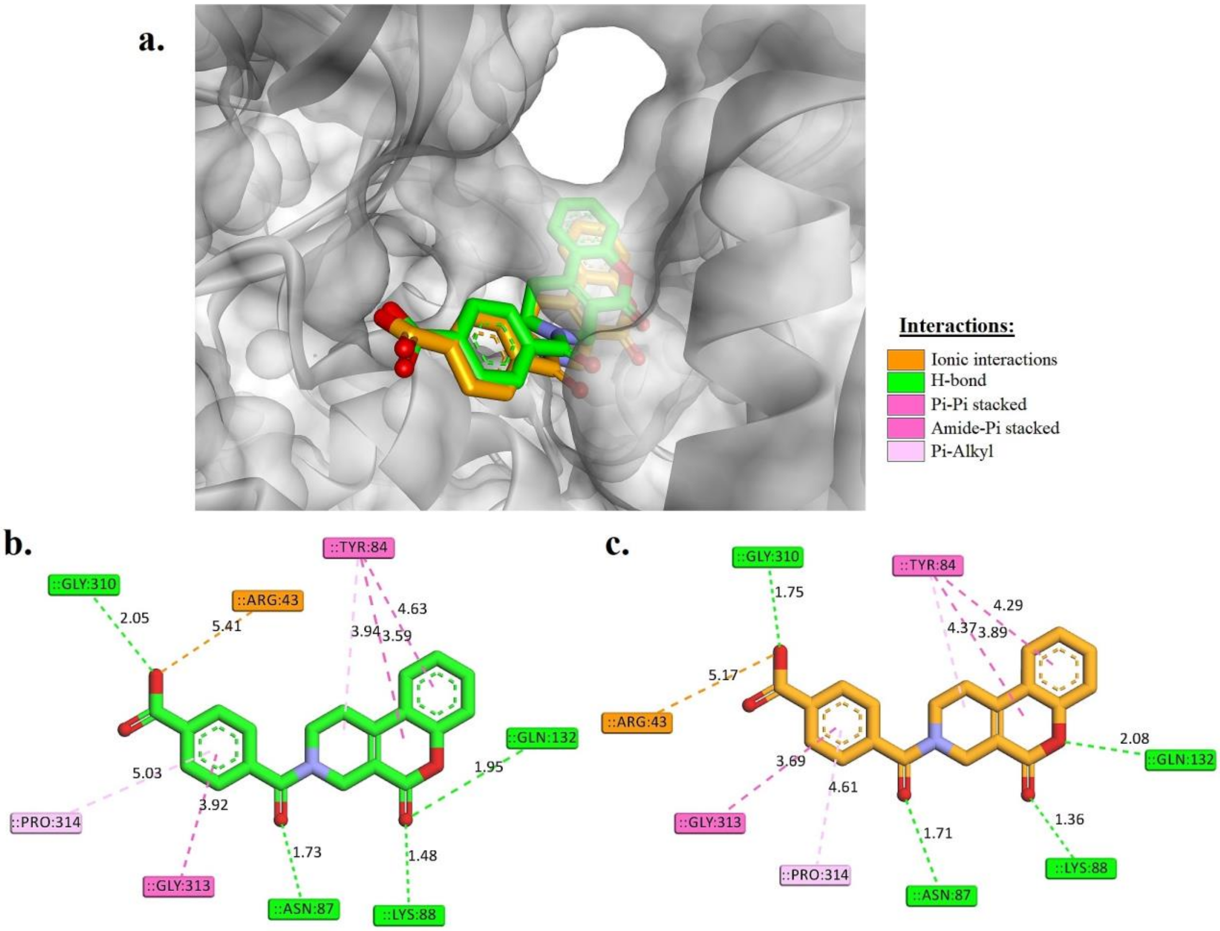

Figure S1. (a.) Superimposition and (b.) 2D interaction analysis of the co-crystallized ligand (DS44960156) (highlighted in green for carbon (C), red for oxygen (O), and navy for nitrogen (N)). (c.) Re-docked ligand (highlighted in orange for carbon (C), red for oxygen (O), and navy for nitrogen (N)) within the crystal structure of the human MTHFD2 enzyme (PDB ID: 6JIB).
